# Fogging and Flight Interception Traps—The Evaluation of Two Methods to Collect Canopy Arthropods

**DOI:** 10.1002/ece3.73276

**Published:** 2026-03-30

**Authors:** Andreas Floren, Martin M. Gossner, Tobias Müller

**Affiliations:** ^1^ Department of Animal Ecology and Tropical Biology, Biocenter University of Würzburg Würzburg Germany; ^2^ Department of Bioinformatics, Biocenter University of Würzburg Würzburg Germany; ^3^ Forest Entomology Swiss Federal Research Institute WSL Birmensdorf Switzerland; ^4^ Department of Environmental Systems Science Institute of Terrestrial Ecosystems, ETH Zürich Zürich Switzerland

**Keywords:** canopy diversity, community analysis, ecosystem characterisation, insecticidal fogging, sampling characteristics, window traps

## Abstract

The forest canopy is a crucial habitat for a wide variety of species. However, it remains difficult to assess due to significant methodological challenges and uncertainties. Flight intercept traps (FITs) and insecticidal knockdown (FOGGING) are among the most widely used techniques for sampling canopy arthropods, yet systematic comparisons of their taxonomic and ecological coverage remain scarce. We sampled arthropods over 3 months in nine forest plots in the Schorfheide‐Chorin Biosphere Reserve, Germany, using both methods on two dominant tree species. A single FIT covered only a small portion of the crown and yielded on average ~100 individuals per month, dominated by Diptera, with a June peak of Coleoptera. In contrast, FOGGING targets entire tree crowns, capturing 1000 individuals on average, providing a broader and more balanced representation of arthropod communities across orders throughout the season. When Coleoptera was used as the target taxon, FOGGING achieved substantially higher sample coverage across families, genera and species than FITs, which primarily collected highly mobile taxa (e.g., Elateridae) and xylobionts (e.g., *Scolytinae*). FITs exhibited higher beta diversity, reflecting the stochastic sampling of rare species. In contrast, FOGGING sampled more consistent communities with lower turnover. Community‐level metrics, including diversity, rank‐abundance structure and trait composition (e.g., body size and functional guilds), also differed significantly between the two methods. Our results suggest that FOGGING provides a more comprehensive representation of the taxonomy and ecology of canopy arthropod communities, offering a stronger basis for ecological conclusions. Our findings are consistent with previous research indicating that the two sampling methods complement each other rather than being interchangeable. This emphasises the importance of carefully tailoring sampling methods and trap types to specific research questions.

## 1 Introduction

Tree crowns harbour an abundant and highly diverse arthropod fauna that is essential for ecosystem functioning. Their influence extends beyond the canopy and interacts with other compartments, such as the soil biota. This highlights the complex interactions within forest ecosystems (Swart et al. 2020; Noriega et al. 2018; Bardgett and Wardle 2010). A striking example of this is the background herbivory by caterpillars. Even outside of outbreak situations, continuous leaf consumption by caterpillars contributes substantially to the supply of nutrients to root‐associated microbial communities (Castaño et al. 2020; Conrad‐Rooney et al. 2020; Hwang et al. 2024) (Canelles et al. 2021; Jactel et al. 2021). Such results illustrate the key role of canopy arthropods in forest ecosystems and the necessity of incorporating them into ecological research, particularly when assessing the impacts of forest management or climate change, as understory communities may respond differently (Leidinger et al. 2019).

The ecological characterisation of canopy arthropods is, however, strongly influenced by the sampling method, as each approach captures distinct fractions of the fauna (Busse et al. 2022; Seibold et al. 2018; Sinclair et al. 2024). This has fuelled debate over how divergent, or complementary, the results from different methods are, particularly between widely used flight interception traps (FITs) and canopy FOGGING (Floren and Schmidl 2008). FITs passively intercept arthropods along their flight paths, mainly capturing highly mobile taxa such as Diptera and Coleoptera (Wildermuth et al. 2023). Operated continuously over weeks, they integrate diurnal weather‐related variations in activity and pool arthropods from multiple canopies depending on trap placement (Kowalski et al. 2011). Less mobile taxa are likely underrepresented. In contrast, FOGGING provides a spatially and temporally well‐defined snapshot of tree‐specific communities (Floren, Horchler, and Müller 2022). Its main advantage lies in the comprehensive and largely unbiased sampling of the ectophytic canopy fauna, collecting individuals approximately in proportion to their true abundance and including highly mobile taxa such as Diptera and Coleoptera. Cryptic species, such as endophytes, bark‐ or cavity‐dwellers and scale insects, are underrepresented. Although often criticised for its presumed ecological impact, studies demonstrate that, when carefully applied, its effects are minimal and confined to a small radius around the studied tree (Floren, Horchler, and Müller 2022; Thunes et al. 2021; Wildermuth, Penanhoat, et al. 2024). Unlike FITs, FOGGING requires favourable weather conditions.

Both FITs and FOGGING have been used to analyse environmental drivers of canopy arthropod communities, but their sampling properties have not yet been systematically compared under standardised conditions. A quantitative evaluation is therefore overdue to guide researchers in selecting the most appropriate method. The German ‘Biodiversity Exploratories’ (Fischer et al. 2010) provided a unique opportunity to compare both methods within the same plots, tree species ( *Fagus sylvatica* and *Pinus sylvestris* ) and year, under standardised field conditions. Specifically, we asked:

1. How do FITs and FOGGING differ in capture efficiency, and how does this influence the representation of arboreal community diversity, structure and seasonal dynamics?
2. Do both methods similarly represent ecologically relevant trait distributions, such as body size or guild composition?
3. How do our results compare to other published studies, and what do they imply for the complementary use of canopy sampling methods in ecological research?

## 2 Methods

### 2.1 Study Area and Sites

The study was conducted in the UNESCO Biosphere Reserve Schorfheide‐Chorin in north‐eastern Germany (13°23′27″–14°08′53″ E/52°47′25″–53°13′26″ N), as part of the long‐term Biodiversity Exploratories program. The post‐glacial moraine landscape is characterised by sandy, acidic soils at 3–140 m a.s.l., with annual precipitation of 500–600 mm and mean temperatures of 8°C–8.5°C (Fischer et al. 2010). Forests are dominated by pine (67%) and beech (28%).

We studied three forest management types: (1) managed, even‐aged pine ( *Pinus sylvestris* ), (2) managed, even‐aged beech ( *Fagus sylvatica* ) and (3) unmanaged beech stands left to succession for more than 20 years prior to sampling. Unmanaged stands showed high structural complexity, while even‐aged beech was more homogeneous, consisting of a single canopy layer and managed in ~120‐year rotations using shelter‐wood systems. Pine stands were harvested in small‐scale clearcuts every 60–80 years, reflecting typical regional silvicultural practice. Pine is not native and occurs mainly on nutrient‐poor soils. Both beech and pine share similar climatic and edaphic conditions. Management intensity was quantified with the continuous Silvicultural Management Intensity index (SMI) (Gossner et al. 2014), which was integrated into PERMANOVA models to account for site differences. The SMI comprises two factors: (1) the risk of stand loss due to hazards such as wind and pests, taking into account information on tree species, stand age and the biomass of living and dead wood and (2) relative stand density.

### 2.2 Study Trees and Sampling Methods

**FIGURE 1:**
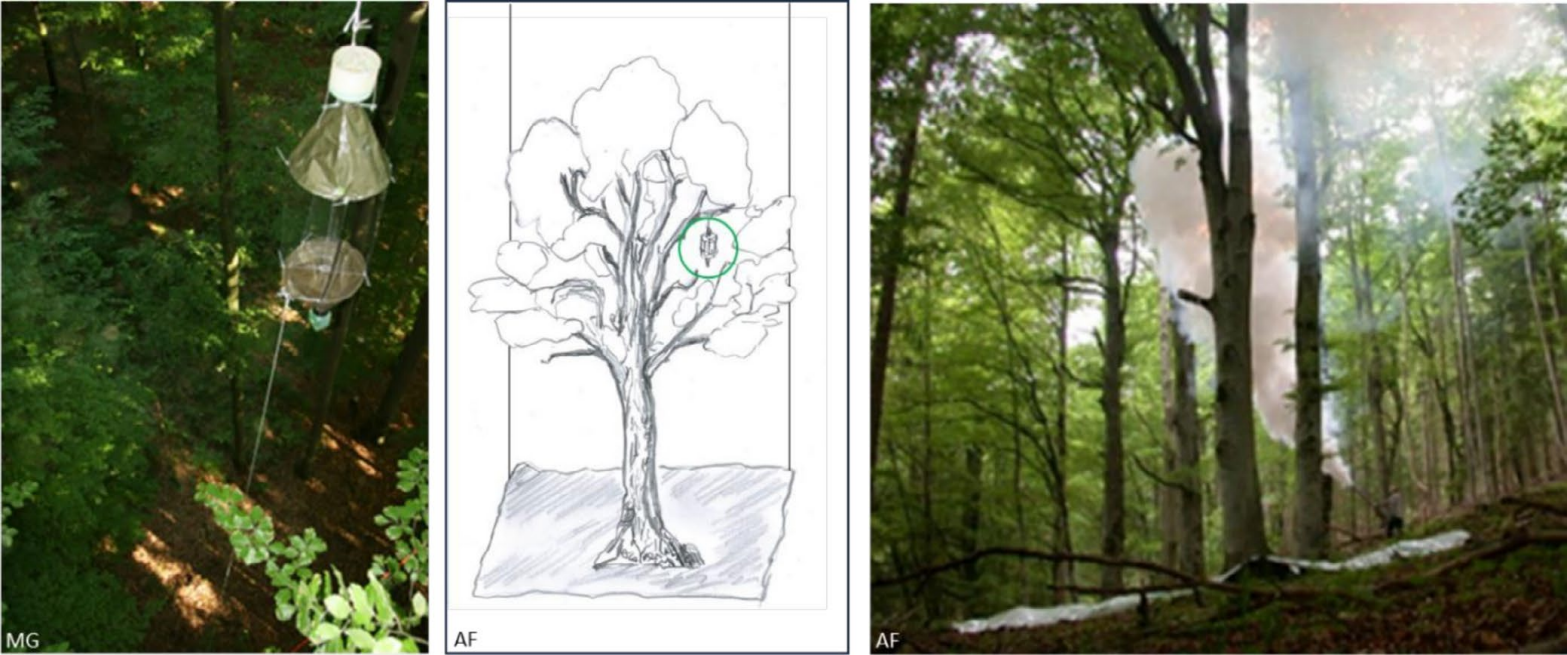
Flight interception traps (left) were installed in two trees per stand and emptied after 4 weeks in June, August and September. Insecticidal FOGGING (right) was conducted in the same plots and months. The central illustration highlights the contrasting sampling volumes of the two approaches: FITs target a highly localised portion of the tree crown (green circle), while FOGGING samples the entire crown. Insects were collected in collecting sheets (grey), delineating the effective canopy sampling volume.

Sampling was conducted in 2010. At each of nine forest stands (five beech, four pine), one flight interception trap (FIT) was installed in the vertical crown centre of two conspecific trees, approximately 100 m apart. FITs consisted of crossed transparent sheets (40 × 60 cm) with funnels leading into collecting jars (Figure 1). The lower jar mainly captured species that showed a fright reaction when they were intercepted by the window, while the upper jar mainly collected phototactic taxa such as Diptera and Hymenoptera (Knuff et al. 2019). Traps were filled with 3% copper‐sulphate solution plus detergent and emptied monthly in June, August and September (Table 1). Samples were preserved in 70% ethanol.

**TABLE 1:** Number of flight interception traps (FITs) and FOGGINGs on *Fagus sylvatica* and *Pinus sylvestris* (in brackets) across the season. Two trees per site were equipped with FITs; two pine FIT samples were lost in September. In June, two trees per site were fogged to capture peak abundance. Three trees could not be fogged due to wildfire risk.

Sample method	Month	*Fagus* (5)	*Pinus* (4)	Sum
FITs	June	10	8	
FITs	August	10	8	
FITs	September	10	6	52
FOGGINGs	June	9	8	
FOGGINGs	August	5	3	
FOGGINGs	September	5	3	33

FOGGING was conducted using natural pyrethrum (1% active substance), which is arthropod‐specific, photodegradable and dissipates rapidly without leaving any residues on the vegetation (Floren, Horchler, and Müller 2022). The warm fog penetrates the full crown, affecting all free‐living arthropods. Applications were performed in the early morning under calm conditions. Each tree was fogged for 5–10 min, and collecting sheets were precisely placed under the crown projection to exclude arthropods from neighbouring trees. After 2 h, all arthropods were collected and preserved in 80% ethanol.

Seasonal coverage was achieved by FOGGING two trees per plot in June when abundance peaked and one new tree per plot in August and September. Within each plot, FOGGING and FITs were conducted on separate trees at least 100 m apart to avoid cross‐contamination. Of the 36 intended FOGGINGs, three could not be performed due to fire risk.

### 2.3 Identification and Trait Classification at Ordinal and Species Level

From all samples Diptera, Coleoptera, Hemiptera, Hymenoptera, Psocoptera, Lepidoptera and Araneae were sorted, separated from all remaining arthropods, and pooled as “Others.” Within Hemiptera, Homoptera (Sternorrhyncha, Auchenorrhyncha) and Heteroptera were analysed separately due to their distinct ecology. Species‐level analyses focused on Coleoptera, which were abundant, diverse in both species and ecological traits. Araneae and Heteroptera were represented by too few adult individuals to allow meaningful analysis. Beetles were identified by specialists (see Acknowledgements). Voucher specimens are deposited in the Bavarian State Collection, Munich, and in the collection of AF. Beetle body size and feeding guild assignments (zoophages, phytophages, xylophages, mycetophages, saprophages) were compiled from the literature (Köhler 2000; Rheinheimer and Hassler 2010; Freude et al. 1964–1983). Differences between FITs and FOGGING were analysed separately for beech and pine.

### 2.4 Statistical Analyses

All analyses were performed in R (R Core Team 2024) using vegan (Oksanen et al. 2022), ade4 (Chessel and Dufour 2012), mgcv (Wood 2017) and Bioconductor packages (Marini et al. 2020). We considered four categorical factors: (1) Trap (FIT, FOGGING), (2) Tree species (*Fagus*, *Pinus*), (3) PlotID (nine sites) and (4) Month (June, August, September).

Community‐level differences between FITs and FOGGING were visualised with empirical cumulative distribution functions (ECDFs). Proportional differences at the order and Coleoptera family level were tested using mixed‐effect logistic regression in a GAM framework, with PlotID as a random effect and binomial/quasibinomial families depending on dispersion. Feeding guild composition was modelled within the same framework, separately for total beetles and xylobiont beetles. Singleton numbers and their discriminatory power between traps were analysed with negative binomial regression, including Species × Trap interactions. Community structure was compared via rank abundance curves, rank positions of dominant taxa, and species overlap using Venn diagrams and UpSet plots. Sampling completeness was assessed by individual‐based rarefaction and extrapolation using iNEXT (Hsieh et al. 2022) and Chao coverage estimates, modelled as Coverage ~ Trap + (1|PlotID). Beetle body‐size distributions were compared by density plots and tested with the Kolmogorov–Smirnov test. Beta diversity was assessed with NMDS (abundance‐based Sørensen index, *k* = 4) and PERMANOVA (adonis2, marginal tests). The model Sørensen beta diversity ~ Tree × Trap + SMI tested the effects of trap type, tree species and silvicultural management intensity. Significance was evaluated using 99,999 permutations. Within‐tree beta diversity was modelled separately for pine and beech as beta diversity ~ Trap + SMI. Guild composition was visualised with box plots and tested with quasibinomial mixed‐effect regression (Guild proportion ~ Trap + (1|PlotID)). Species similarity among guilds was quantified with the Jaccard index. All *p*‐values were adjusted for multiple testing using (Benjamini and Hochberg 1995), with significance indicated as *p < 0.05; **p < 0.01; ***p < 0.001.

## 3 Results

### 3.1 Order Composition

Of the 37,776 arthropods collected, 89.4% came from FOGGING (33,763 individuals) and 10.6% from FITs (4013 individuals). On average, FITs captured 77 specimens per trap, whereas FOGGING collected 1023 individuals per tree. Method‐specific differences strongly altered community composition on both *Fagus* and *Pinus*, resulting in different taxonomic representations. Diptera dominated all samples throughout the season, with higher numbers and relative proportions in FITs, particularly on *Pinus* (FITs 60% in FITs vs. 21.8% in FOGGING; on *Fagus*: 51.5% vs. 42.1%). Arthropod abundance peaked in June, when Coleoptera ranked second and were proportionately much higher in FITs (*Fagus*: 25.7% vs. 6.1%; *Pinus*: 26.5% vs. 9.5%). All other taxa were collected in low numbers in FITs (Table 2).

**TABLE 2:** Arthropod abundance and relative proportions by order on *Fagus sylvatica* (Fs) and *Pinus sylvestris* (Ps). Arthropod abundance peaked in June, with pronounced differences between flight interception traps (FITs) and insecticidal fogging (FOGGING) across sampling months. Shading indicates variation in the relative proportion of each order within samples (light shading: 18%–27%; dark shading: > 40%).

	Jun:Fs:FIT	Jun:Fs:FOG	Jun:Ps:FIT	Jun:Ps:FOG	Ags:Fs:FIT	Ags:Fs:FOG	Ags:Ps:FIT	Ags:Ps:FOG	Spt:Fs:FIT	Spt:Fs:FOG	Spt:Ps:FIT	Spt:Ps:FOG	Sum
Diptera	491	7746	352	2085	226	428	343	219	215	2353	563	218	15,239
	*47%*	*44.2%*	*41%*	*25.4%*	*50.3%*	*26%*	*61.5%*	*18.8%*	*57.2%*	*56.2%*	*77.4%*	*21.1%*	
Homoptera	51	3880	43	1871	9	125	24	227	5	111	22	250	6618
	*4.9%*	*22.2%*	*5%*	*22.8%*	*2%*	*7.6%*	*4.3%*	*19.5%*	*1.3%*	*2.7%*	*3%*	*24.2%*	
Hymenoptera	87	1620	86	1633	42	408	88	186	30	505	66	186	4937
	*8.3%*	*9.3%*	*10%*	*19.9%*	*9.4%*	*24.8%*	*15.8%*	*16%*	*8%*	*12.1%*	*9.1%*	*18%*	
Coleoptera	268	1073	228	780	30	118	23	85	19	614	16	126	3380
	*25.7%*	*6.1%*	*26.5%*	*9.5%*	*6.7%*	*7.2%*	*4.1%*	*7.3%*	*5.1%*	*14.7%*	*2.2%*	*12.2%*	
Heteroptera	7	1031	12	612	33	35	5	29	17	37	4	22	1844
	*0.7%*	*5.9%*	*1.4%*	*7.4%*	*7.3%*	*2.1%*	*0.9%*	*2.5%*	*4.5%*	*0.9%*	*0.6%*	*2.1%*	
Psocoptera	35	688	31	547	4	103	5	180	2	49	1	31	1676
	*3.4%*	*3.9%*	*3.6%*	*6.7%*	*0.9%*	*6.2%*	*0.9%*	*15.4%*	*0.5%*	*1.2%*	*0.1%*	*3%*	
Lepidoptera	27	576	17	361	18	110	2	106	2	39	3	27	1288
	*2.6%*	*3.3%*	*2%*	*4.4%*	*4%*	*6.7%*	*0.4%*	*9.1%*	*0.5%*	*0.9%*	*0.2%*	*2.6%*	
Araneae	60	426	29	147	55	251	59	63	79	363	45	122	1699
	*5.7%*	*2.4%*	*3.4%*	*1.8%*	*12.2%*	*15.2%*	*10.6%*	*5.4%*	*21%*	*8.7%*	*6.2%*	*11.8%*	
Others	18	470	61	185	32	70	9	71	7	115	7	50	1095
	*1.7%*	*2.7%*	*7.1%*	*2.3%*	*7.1%*	*4.2%*	*1.6%*	*6.1%*	*1.9%*	*2.7%*	*1%*	*4.8%*	
Sum	1044	17,510	859	8221	449	1648	558	1166	376	4186	727	1032	37,776

*Note:* Shading indicates the relative proportion of each order within the samples.

Diptera and Coleoptera together accounted for 74% of all arthropods on *Fagus* sampled with FITs, compared with 51.8% in FOGGING. On *Pinus*, the corresponding values were 72.7% and 35.7%. By contrast, Homoptera and Heteroptera were consistently more abundant in FOGGING (June *Fagus*: FIT 4.9%, FOGGING 22.2%; *Pinus*: FIT 5.0%, FOGGING 22.8%). Seasonal declines were more pronounced in the FITs, which yielded only 30 beetles in August and 19 in September, compared to hundreds in the FOGGING (up to 14.7% beetles in *Fagus* and 12.2% in *Pinus* in September; Figure S1). Only parasitic Hymenoptera and Araneae showed little seasonal variation. Due to the low FIT catch rates later in the season, subsequent analyses focused on June.

**FIGURE 2:**
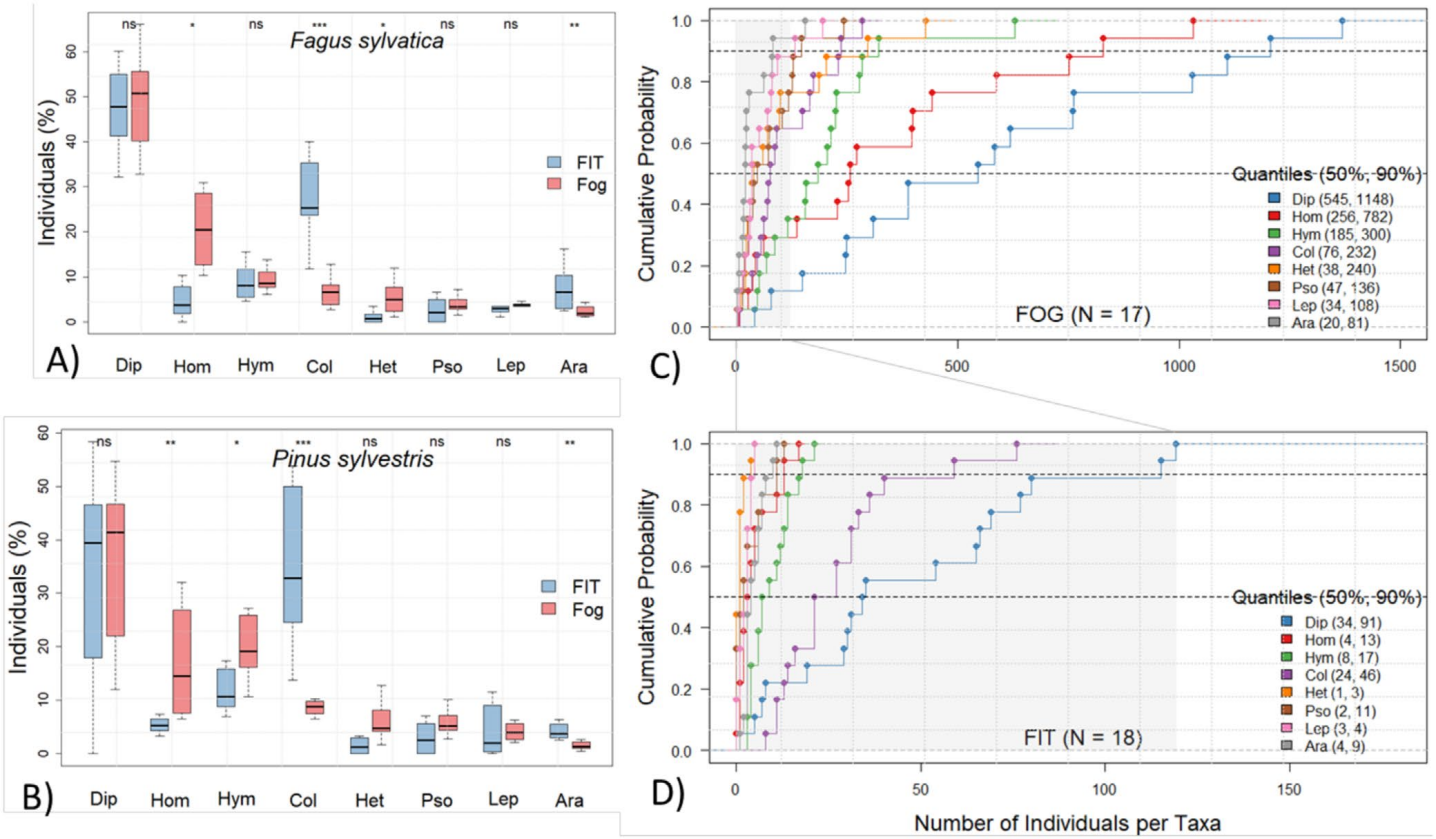
Boxplots show the proportional distribution of major arthropod orders collected by flight interception traps (FITs) and insecticidal fogging (FOGGING) on *Fagus* (A) and *Pinus* (B) in June. Significance levels are indicated above each comparison. Empirical cumulative distribution functions are shown for FOGGING (C) and FITs (D). Data from both tree species were combined, as order‐level distributions did not differ significantly. Sample sizes are given below. The *x*‐axis represents arthropod abundance per taxon, and the *y*‐axis shows the cumulative probability. Points denote individual samples; dashed lines mark the 50% and 90% quantiles (values in brackets in the legend). Shaded areas indicate different scale ranges for FITs and FOGGING. Ara, Araneae; Col, Coleoptera; Dip, Diptera; Het, Heteroptera; Hom, Homoptera; Hym, parasitic Hymenoptera; Lep, Lepidoptera; Pso, Psocoptera. Significance indicated as *p < 0.05; **p < 0.01; ***p < 0.001.

In June, order composition on *Fagus* and *Pinus* was similar within methods, but relative abundances differed strongly between FITs and FOGGING (Figure 2A,B). FITs contained significantly higher proportions of Coleoptera and Araneae, whereas Homoptera dominated more strongly in FOGGING. Diptera, parasitoid Hymenoptera and other orders showed only weak or no tree‐specific differences. Empirical cumulative distribution functions (ECDFs) further illustrated these contrasts (Figure 2C,D). For instance, 50% of FOGGING samples contained ≥ 545 Diptera and 90% ≤ 1148, while the low counts from the FITs compressed the *x*‐axis, producing steep cumulative curves. Overall, FOGGING collected a broader range of taxa in high abundances, whereas FITs yielded substantial numbers only for Diptera and Coleoptera. The shaded areas in the ECDFs emphasise the scale differences and highlight the significant divergence in community composition between methods.

### 3.2 Coleoptera Communities

From the 3277 beetles (277 species), we analysed the June samples in detail, comprising 512 individuals (110 species) from FITs and 1752 specimens (162 species) from FOGGING. Sample coverage was higher in FOGGING on *Fagus* (0.84 vs. 0.62, *p* < 0.05), whereas differences on *Pinus* were smaller and not significant. Overall, FOGGING collected roughly twice as many families, genera and species and four times as many individuals on *Fagus* (Figure S1). Differences were smaller on *Pinus* but still 50% higher in FOGGING. UpSet plots revealed significant differences in community composition, with FOGGING uniquely detecting more taxa at all hierarchical levels (Figure S2). Beetle families showed clear method‐specific biases. FITs were dominated by Elateridae (36.2% and 44.2% of individuals on *Fagus* and *Pinus* and 17% and 20.5% of species, respectively), whereas their proportion in FOGGING was below 10%. Conversely, Curculionidae dominated the FOGGING samples (42%–48% of individuals), but were less abundant in the FITs, except for the bark beetles (*Scolytinae*), which were significantly overrepresented in the FITs (22%–33% of individuals and 15%–18% of species; see Figure S3).

**FIGURE 3:**
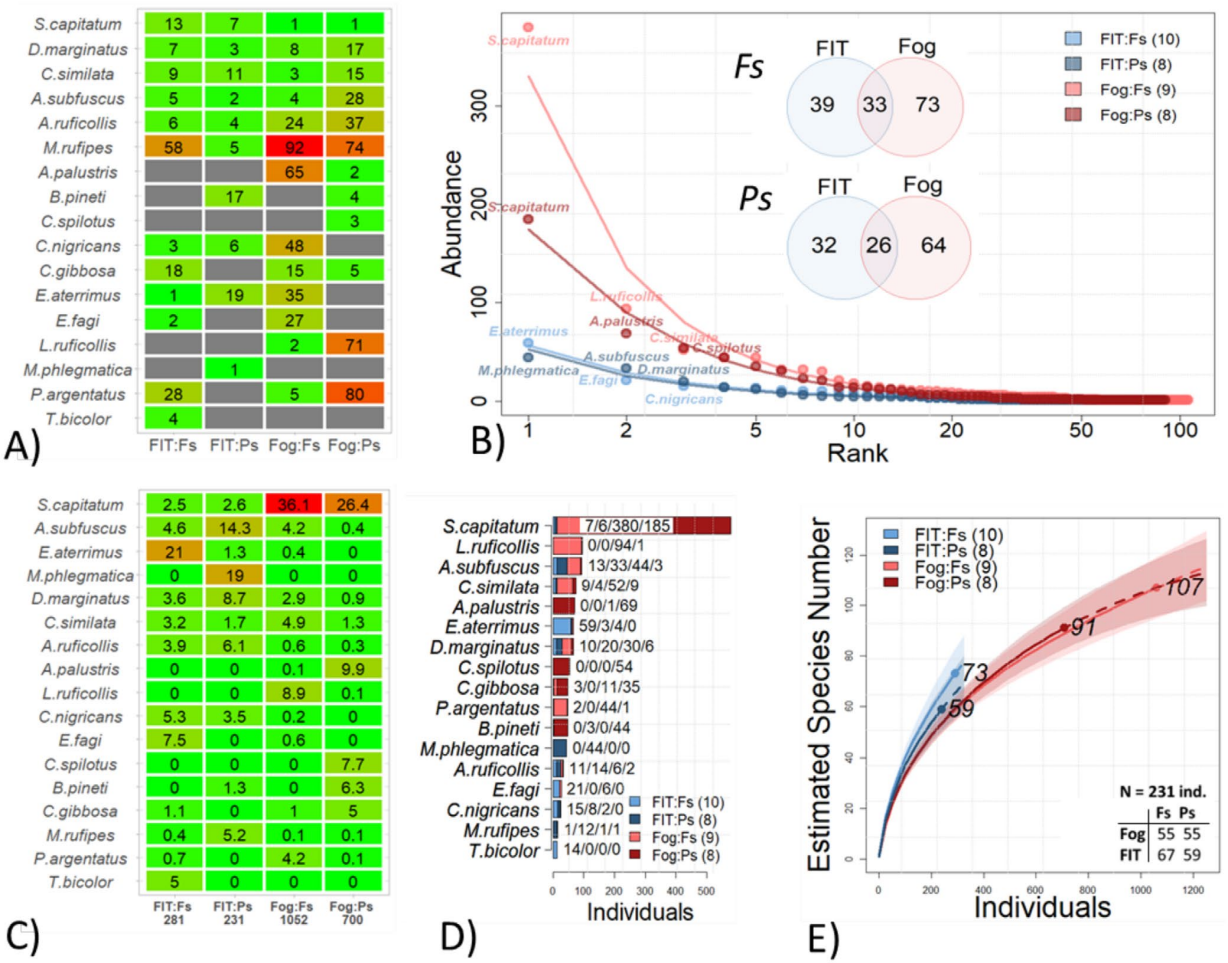
Community diversity and structure of beetles collected by FITs and FOGGING. (A) Heatmap of rank positions for the 17 most common species on *Fagus* (Fs) and *Pinus* (Ps); grey = absent species. (B) Rank abundance curves show similar structures in FITs but clear tree‐specific separation in FOGGING. Venn diagrams illustrate low species overlap between methods. (C) Table and (D) stacked bar plot display major differences in relative proportions and counts; totals below (C). (E) Individual‐based rarefaction curves indicate higher standardised diversity in FITs (231 individuals) due to greater evenness. Full species annotation is provided in Table S1.

Community composition also differed significantly between methods (Figure 3). Although rank abundance curves showed similar structures across tree species in the FITs, distinct tree‐specific patterns were found in FOGGING. Species overlap between methods was low (roughly 22%), with many species exclusive to one method (50% on *Fagus* in FOGGING vs. 27% and 53% on *Pinus* vs. 23%). For example, *Athous subfuscus* and *Ectinus atterimus* (Elateridae) were common in FITs, while *Strophosoma capitatum* and *Phyllobius argentatus* (Curculionidae) dominated in FOGGING. These contrasts directly influenced diversity estimates. FITs exhibited higher evenness and consequently higher standardised diversity by underrepresenting dominant taxa. In contrast, FOGGING produced greater species constancy (significant for *Fagus*, *p* < 0.001) and detected more new species per sample (Figure S4), although neither method reached asymptotic richness in accumulation curves.

**FIGURE 4:**
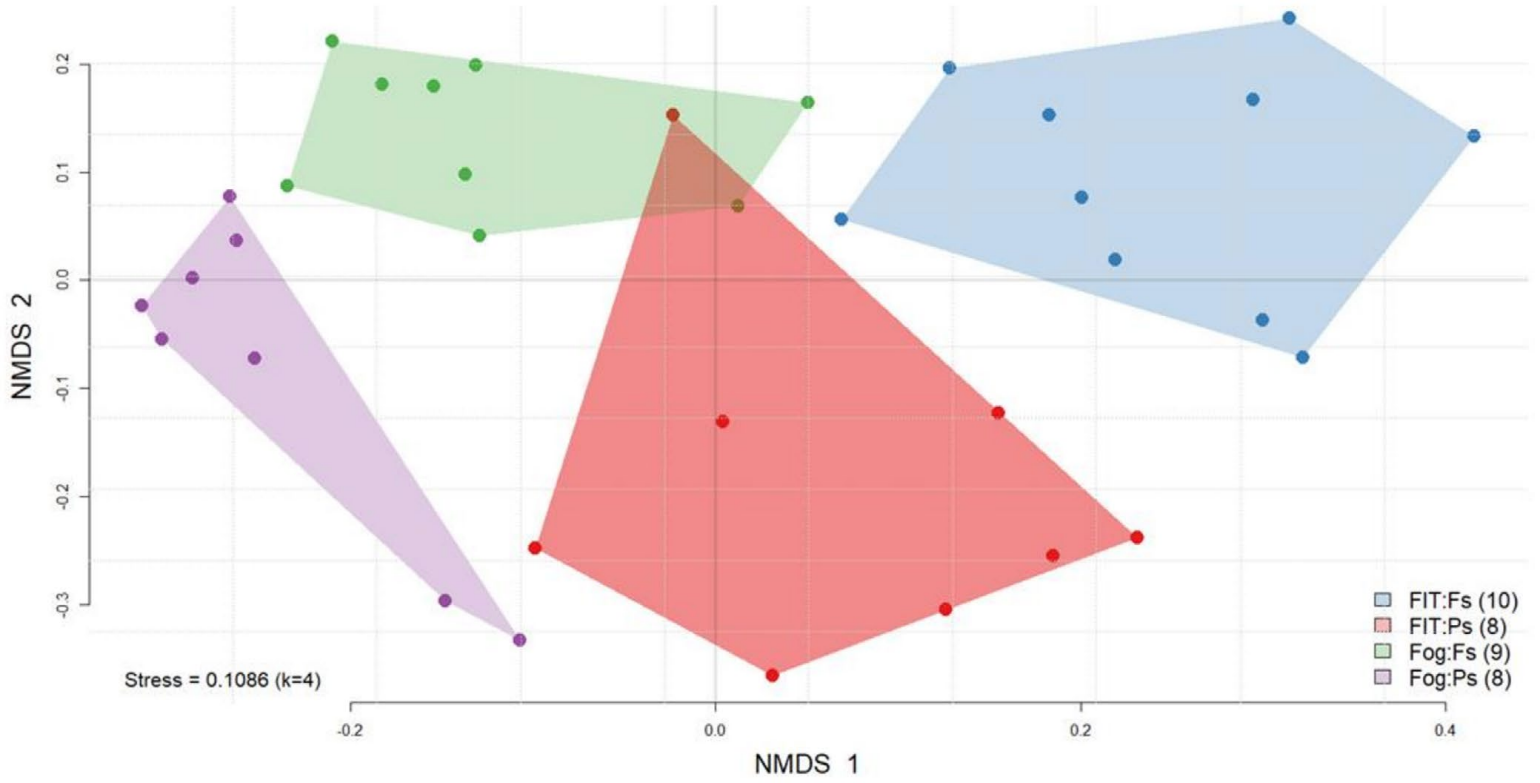
NMDS ordination of beetle assemblages in June based on Sørensen dissimilarity. Axis 1 separates FITs from FOGGING, axis 2 distinguishes tree species. Both factors were highly significant in a PERMANOVA. Larger convex hulls in FITs illustrate greater variability than in FOGGING. The legend shows sample numbers for *Fagus* and *Pinus* in brackets.

NMDS ordinations revealed complete separation among all samples, reflecting strong and highly significant differences between methods and tree species (PERMANOVA; Figure 4; Table S2). Axis 1 clearly distinguished FITs from FOGGING, while axis 2 separated *Fagus* from *Pinus*. Management effects (SMI index) were significant only when including the Tree × Trap interaction, indicating method‐specific representation of site variation. Separate analyses confirmed significant differences between sampling methods within each tree species. Anderson beta diversity was consistently higher in FITs (0.49 for both species) compared with FOGGING (0.26 for *Fagus*, 0.39 for *Pinus*), demonstrating that FITs captured more heterogeneous communities.

**FIGURE 5:**
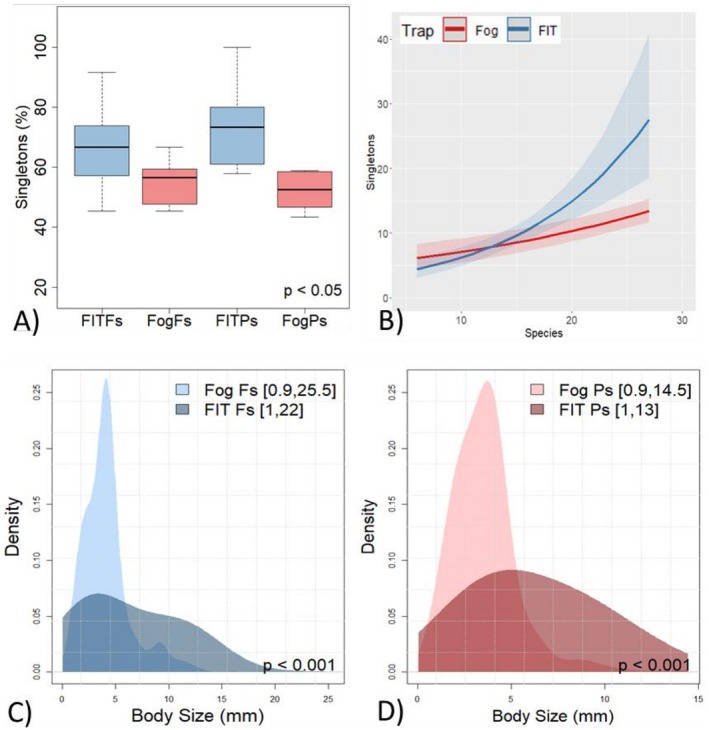
(A) FITs captured significantly more singleton beetles than FOGGING, independent of tree species. (B) Relationship between singletons and total species richness differed between methods (C, D). Density plots show distinct body size distributions between methods, but not between tree species (ranges in brackets). Fs, *Fagus sylvatica* , Ps, *Pinus sylvestris* .

FITs collected significantly higher proportions of singletons compared to FOGGING on both *Fagus* and *Pinus* (*p* = 0.049; Figure 5A, Table S3). Singleton numbers increased with species richness in both methods, but the relationship was linear in FOGGING and exponential in FITs (*p* < 0.01; Figure 5B). Body size distributions were independent of tree species but were strongly method‐specific (KS‐test, *p* < 0.001). FOGGING captured more small‐bodied beetles, whereas FITs sampled relatively fewer small species (Figure 5C,D).

**FIGURE 6:**
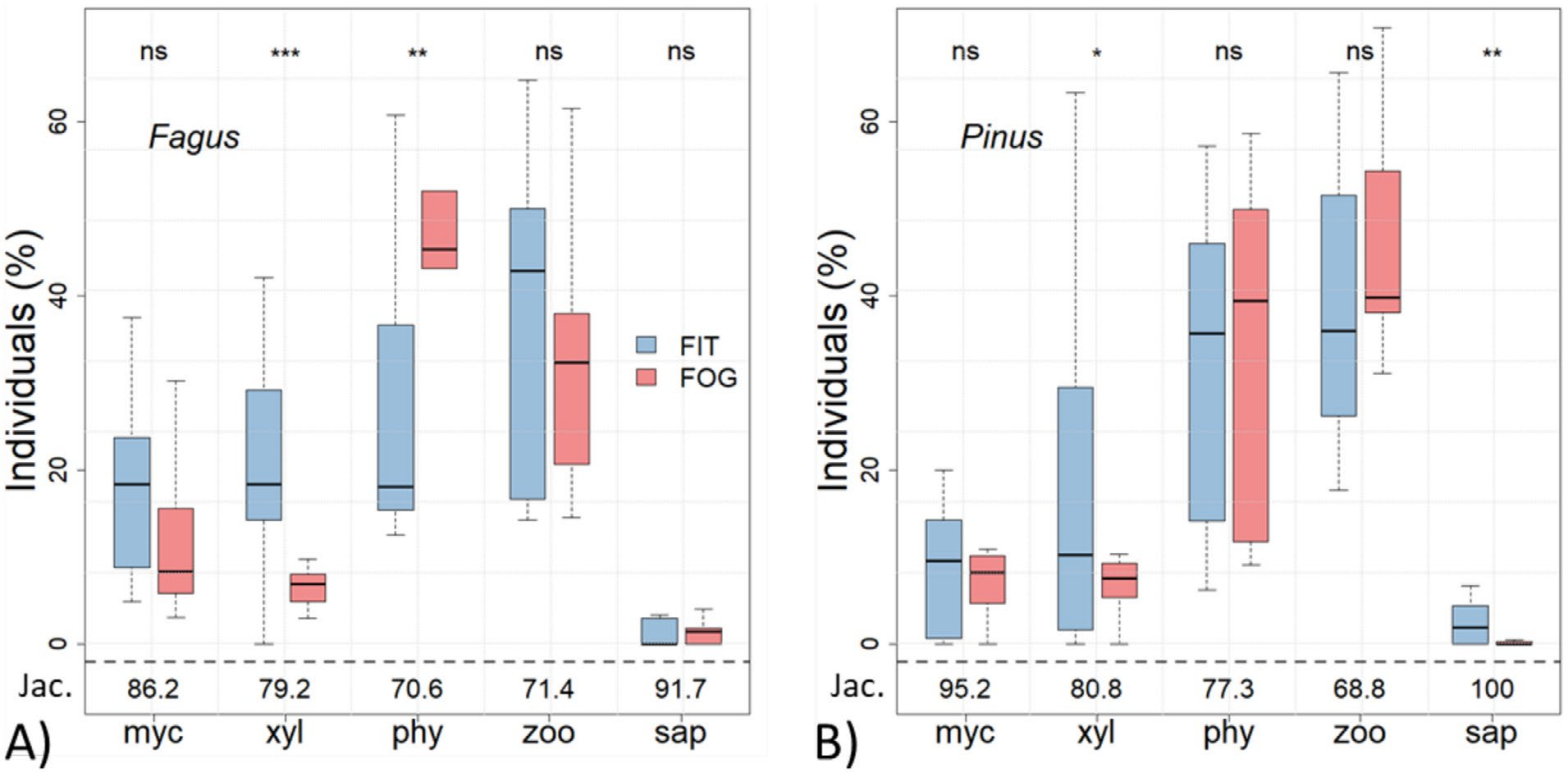
Box plots show significant differences in beetle feeding guild composition between FITs and FOGGING on beech and pine trees. (A) On *Fagus*, FITs yielded more xylophages and fewer phytophages; (B) on *Pinus*, only xylophages differed weakly. Jaccard dissimilarities below the plots consistently indicate low species overlap between methods. myc, mycetophages; phy, phytophages; sap, saprophages; xyl, xylophages; zoo, zoophages. Significance indicated as *p < 0.05; **p < 0.01; ***p < 0.001.

Feeding guild composition differed significantly between FITs and FOGGING but not between tree species (Figure 6). FITs captured proportionally more xylophages, while phytophages dominated in FOGGING. This pattern was most pronounced on *Fagus* and weaker though consistent on *Pinus*. Other guilds showed no significant differences between methods. Jaccard dissimilarities exceeded 70% for most guilds, highlighting the low degree of overlap between methods. FITs also collected a significantly higher proportion of xylobiont beetles (50.2% on *Fagus*, 51.1% on *Pinus*) compared to FOGGING (24.8% and 25.3%, respectively; Figure S5, Tables S4 and S5).

## 4 Discussion

### 4.1 Comparative Sampling Characteristics of FITs and FOGGING

Flight interception traps and FOGGING represent fundamentally different approaches for sampling arboreal arthropods, resulting in distinct community profiles. This was already evident in the overall catch size. In our comparative study, a single FOGGING collected an average of 1023 individuals per tree, compared to 77 individuals in FITs. FITs passively intercept arthropods along a narrow canopy section and accumulate individuals over several weeks. In contrast, FOGGING provides an instantaneous, spatially comprehensive snapshot of the canopy fauna (Floren, Horchler, and Müller 2022). These differences imply that FITs primarily capture activity‐based subsets of the canopy fauna (Gruppe et al. 2008) rather than complete communities. While it is not surprising that FOGGING collects more arthropods than FITs, the magnitude of this difference was unexpected, highlighting the contrasting spatial and temporal scales covered by the two methods. Consequently, FITs are typically pooled across months to compensate for their low sampling efficiency (Burner et al. 2021; Gillespie et al. 2017; Wildermuth, Hagge, et al. 2024).

### 4.2 Order Composition and Seasonal Variation

Our study showed that FITs primarily collected mobile taxa such as Diptera, Hymenoptera and Araneae, whereas FOGGING produced a broader and more balanced taxonomic representation. Diptera dominated in both methods, however (Knuff et al. 2019; Stiegel et al. 2020; Wildermuth, Hagge, et al. 2024). Seasonal dynamics affected the two methods differently. Coleoptera in the FITs were almost exclusively captured in June, with an average of 106 beetles collected per trap, equivalent to three to four specimens per day. FOGGING consistently captured larger and compositionally more balanced communities throughout the season (Stork and Hammond 1997; Simandl 1993; Southwood et al. 2005; Horstmann and Floren 2008; Floren, Linsenmair, and Müller 2022), including the secondary beetle peak in September that FITs failed to detect. After June, arthropod abundance declined sharply in the FITs, increasing the influence of stochastic captures. Hence, FITs provide limited value for assessing seasonal turnover but remain suitable for tracking the activity patterns of mobile taxa over time.

FIT efficiency is further shaped by microhabitat variables such as trap orientation and canopy structure; for instance, south‐west‐oriented FITs collected significantly more individuals than north‐east‐facing ones (Floren, own data), which shows that trap position is crucial. FOGGING circumvents such biases by sampling the entire crown volume, including less mobile and brachypterous species, thereby providing a more representative, spatially explicit snapshot of the tree‐specific arthropod community (Floren, Horchler, and Müller 2022).

### 4.3 Beetle Communities

Beetle assemblages sampled in June differed significantly between methods. FOGGING exhibited consistently higher taxonomic coverage across both tree species, identifying twice as many families and genera on *Fagus* and about 50% more on *Pinus*, despite markedly lower sampling effort. FITs displayed higher within‐sample beta diversity and larger singleton proportions, indicating stochastic and spatially heterogeneous captures, while FOGGING produced more consistent and reproducible community profiles. Species overlap between methods was low (< 25%), even among frequent species, confirming that each method targeted distinct subsets of the canopy fauna.

The low efficiency and high sample heterogeneity likely limit functional analyses based on FITs, including food webs and above‐ and below‐ground energy transfer. In contrast, Potapov et al. (2024) were able to demonstrate the consistency of energy fluxes in a tropical rainforest using time‐repeated fogging data. Functional composition differed markedly between methods: FITs preferentially captured highly mobile, common forest beetles, most notably Elateridae and other xylobiont taxa, with *Scolytinae* (bark beetles) being the most frequent. Under outbreak situations, they can account for 40% of all beetles (Wildermuth, Hagge, et al. 2024; Burner et al. 2021). In contrast, FOGGING captured more phytophagous taxa, especially Curculionidae, the dominant canopy beetle family in Central Europe (Floren, Linsenmair, and Müller 2022). This pattern was also confirmed in numerous investigations of tropical rainforests (e.g., Floren et al. 2020; Kasmiatun et al. 2023).

### 4.4 Functional Implications

The methodological contrasts between FITs and FOGGING have important implications for interpreting community diversity and structure. Although both methods discriminated *Fagus* and *Pinus* based on their beetle assemblages, only FOGGING could consistently resolve structural differences. The disproportionately high number of singletons in FITs inflates alpha diversity estimates but also reflects their greater stochasticity. This is one reason why singletons and outbreak taxa, such as bark beetles, are often excluded from community analysis (Gillespie et al. 2017; Wildermuth, Hagge, et al. 2024). Body size distributions also differed significantly between methods, with FOGGING capturing more smaller beetles. This suggests that the two methods provide complementary insights into species interaction strength and community structure (Kalinkat et al. 2015).

Guild composition also differed significantly. FITs overrepresented xylophagous beetles, whereas FOGGING revealed the dominance of phytophages. This makes FITs valuable for studying xylobiont taxa but less suitable for comprehensive functional analyses. Differences in guild composition among forests have been linked to forest management intensity (Burner et al. 2021; Wildermuth, Hagge, et al. 2024; Vergara et al. 2024; Floren, Horchler, and Müller 2022; Pedley et al. 2014). Although our sample size did not allow for a detailed evaluation of management effects, PERMANOVA analyses incorporating the SMI index revealed distinct responses to management depending on the method employed. These results suggest that the effects of management may manifest differently depending on sampling design.

### 4.5 External Validation and Conclusion

The differences observed in the collection methods are closely comparable to the results of large‐scale studies from European forests, which have consistently shown that FITs overrepresent common, mobile forest species, particularly Elateridae and xylobionts. In contrast, FOGGING more accurately captures the true diversity, including the dominance of phytophagous canopy species (Burner et al. 2021; Gillespie et al. 2017; Floren, Linsenmair, and Müller 2022; Floren et al. 2014; Weisser et al. 2023).

The consistency of the results across different forest types and regions strongly suggests that they are an inherent feature of sampling procedures. In conclusion, our comparative analysis demonstrates that FITs and FOGGING capture fundamentally different statistical populations of canopy arthropods. The fact that different methods collect different species is an inherent characteristic of the various sampling methods (Basset et al. 1997). Although FOGGING provides a comprehensive and spatially explicit representation of the main canopy taxa, FITs predominantly sample highly mobile, large‐bodied beetles, with a bias towards xylobiont species. However, while both methods are complementary in terms of species detection, they can lead to opposing ecological interpretations of diversity patterns, functional structure and spatial organisation. This underscores the contrasting ways in which FITs and FOGGING characterise canopy communities. Increasing FIT numbers can improve statistical power but cannot compensate for their intrinsic selectivity. Therefore, the two methods should be regarded as complementary rather than interchangeable. FIT‐derived data should not be used to infer overall canopy diversity or functional organisation without explicit consideration of their sampling bias. Nonetheless, FITs remain valuable tools when the research focuses on xylobiont taxa or when FOGGING is logistically or ethically constrained.

## Author Contributions

A.F. and M.M.G. designed the experiments. A.F. conducted the fogging and M.M.G. was responsible for the FITs, including data aquisition and curation. T.M. developed the mathematical models and undertook the statistical analyses with A.F.; A.F. led the writing of the manuscript. M.M.G. secured the funding. All authors contributed critically to the drafts and gave final approval for publication.

## Funding

This work was supported by Swiss National Science Foundation SNSF (SNF 310030E‐173542/1) and Deutsche Forschungsgemeinschaft (WE 3081/21‐1; WE 3018/9‐1; WI 1816/9‐1).

## Conflicts of Interest

The authors declare no conflicts of interest.

## Supporting information


**Figure S1:** Details on family, genus, species composition of beetle communities.
**Figure S2:** UpSet plots for family, genus, species composition of beetle communities.
**Figure S3:** Proportional on beetle distribution on the family level.
**Figure S4:** Sample based rarefaction curves for beetles collected by FITs and FOGGING.
**Figure S5:** Marginal effects of fitted mixed effect models for xylobiont beetles.
**Table S1:** Species abundance distribution of the most common beetles in FITs and FOGGING.
**Table S2:** Results of beta diversity modelling.
**Table S3:** Result of modelling singleton proportions.
**Table S4:** Guild composition of whole beetle communities and of xylobiont beetles alone.
**Table S5:** Result of modelling xylobiont beetle proportions.
**Raw Data:** All the required data are uploaded as the Supporting Information.

## Data Availability

All the required data are uploaded as the Supporting Information.
