# Supplementary material for: Fogging and Flight Interception Traps—The Evaluation of Two Methods to Collect Canopy Arthropods
Source: Ecol Evol. 2026 Mar 30;16(4):e73276. doi: 10.1002/ece3.73276 (PMC13107268; doi:10.1002/ece3.73276)

**Figure S1:** (A) Boxplots show that FOGGING consistently collected significantly higher numbers of Coleoptera families, genera, species, and individuals from both *Fagus* and *Pinus* during June, August, and September. (B) Bar plots summarize the corresponding results for the aggregated dataset. (C) Modelled distributions indicate significant differences for all metrics except “Genera” on *Pinus*. Owing to the limited number of samples and low arthropod abundance, representation of beetle communities on *Pinus* was less robust. Overall, the data illustrates pronounced differences between the assemblages captured by the two trapping methods.

**
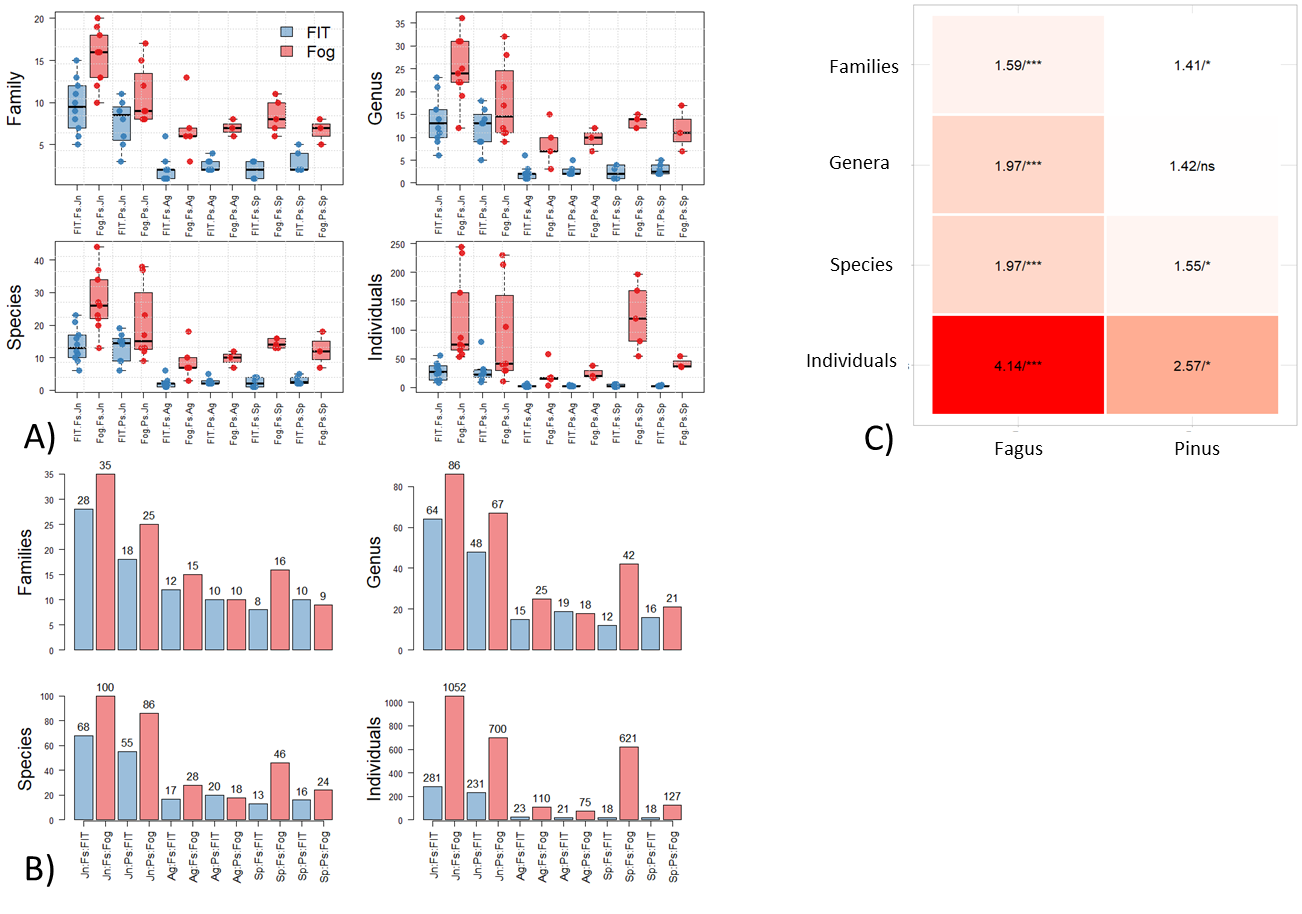
**

**Figure S2:** UpSet plots illustrate high beta diversity between the Coleoptera assemblages collected by the FITs and FOGGING at the family, genus, and species level for *Fagus* and *Pinus* in June. For example, nine beetle families were exclusively fogged from *Fagus*, but only three families were found in the FITs. With increasing taxonomic level, the differences become more pronounced, showing a decrease in taxa in the FITs for both tree species. Samples were sorted according to the total number of families.

**
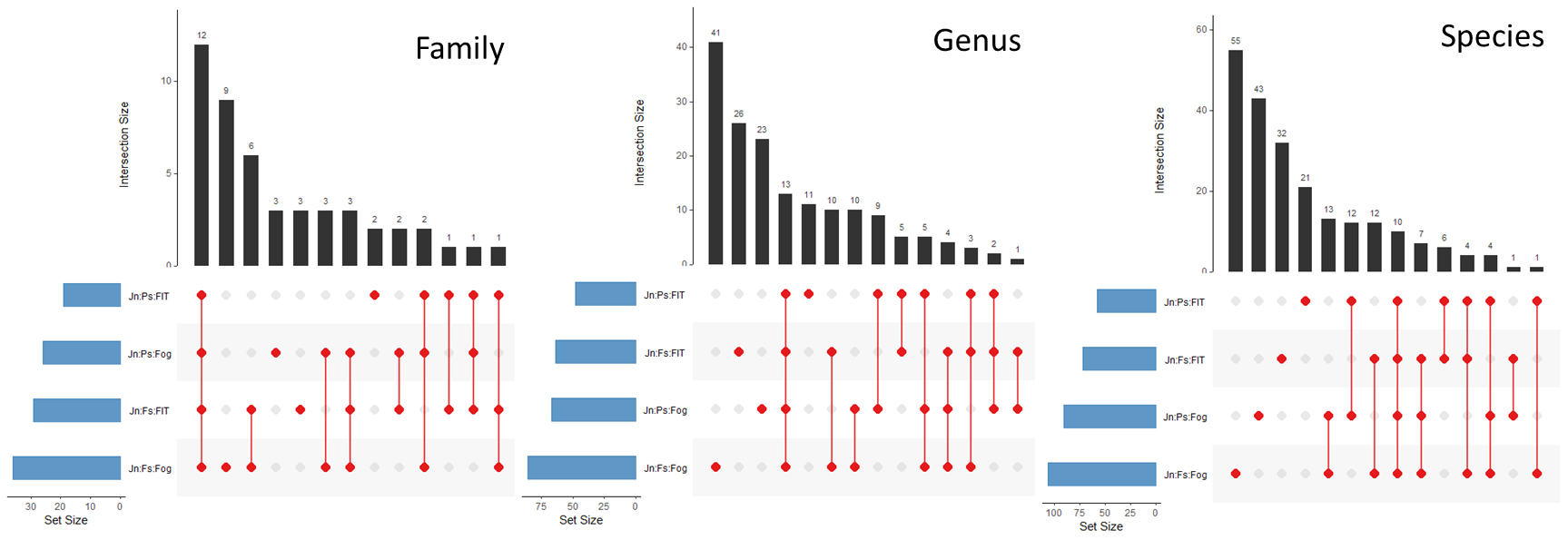
**

**Figure S3:** A) Proportional distribution of individuals and B) species numbers of the ten most abundant beetle families showed significant tree-specific and trap-specific differences between FITs and FOGGINGs.


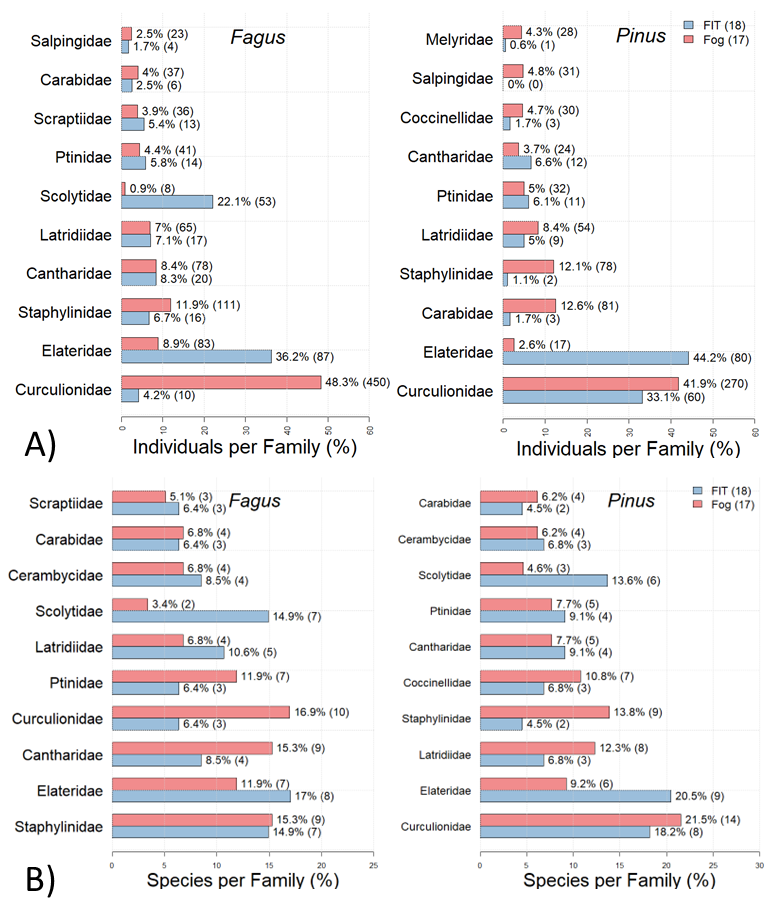


**Figure S4:** Sample-based rarefaction curves show a higher number of new species collected with each FOGGING than with each FIT, without distinguishing tree species on a subsample of eight trees. Fs = *Fagus sylvatica*, Ps = *Pinus sylvestris*; number of samples in brackets.


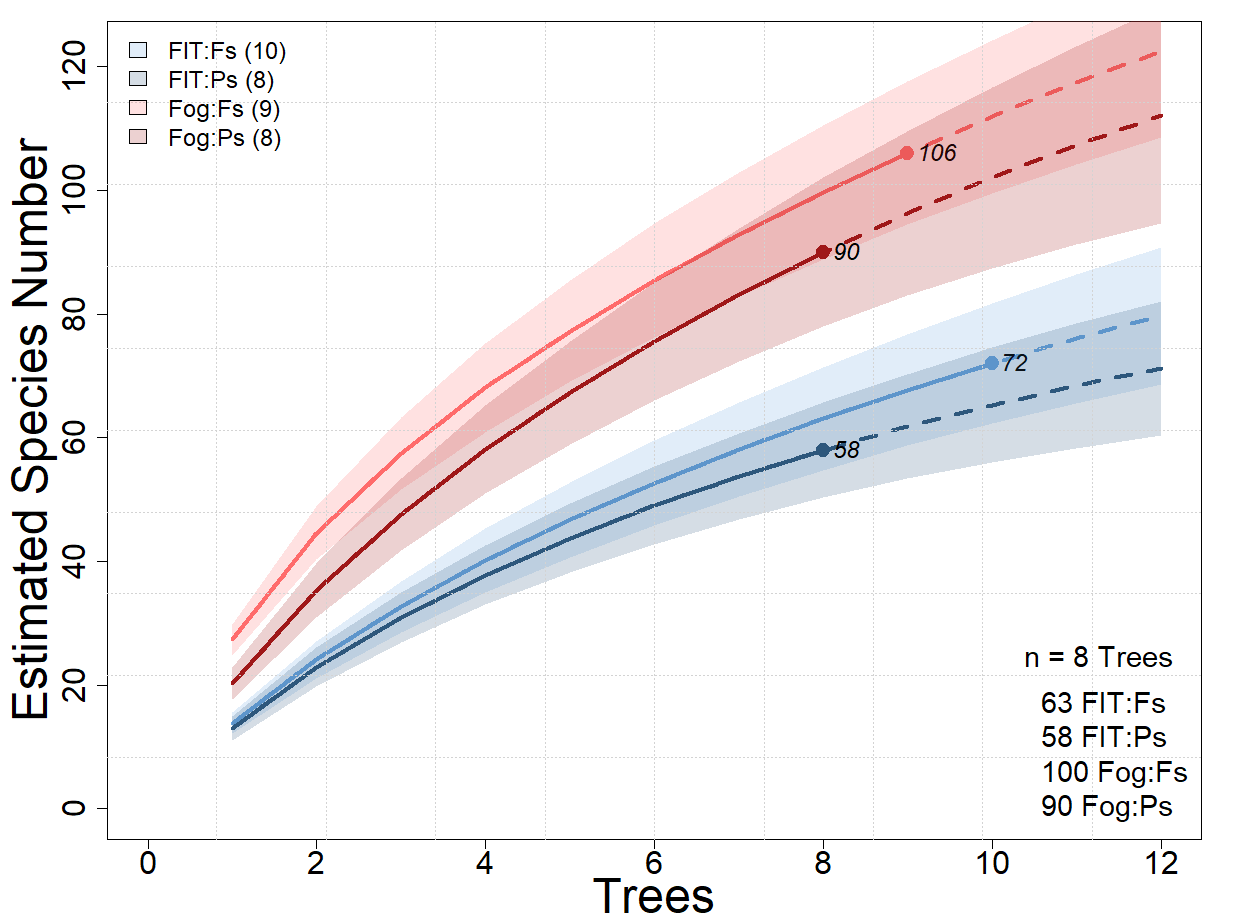


**Figure S5:** Visualisation of marginal effects of fitted mixed effect models for xylobiont beetle individuals and species collected from Fagus sylvatica and Pinus sylvestris and traps in June.


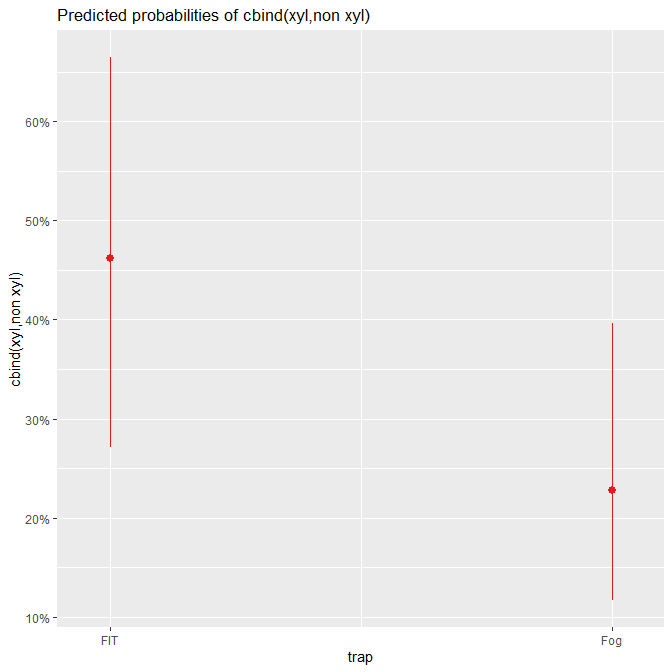


*Fagus individuals*


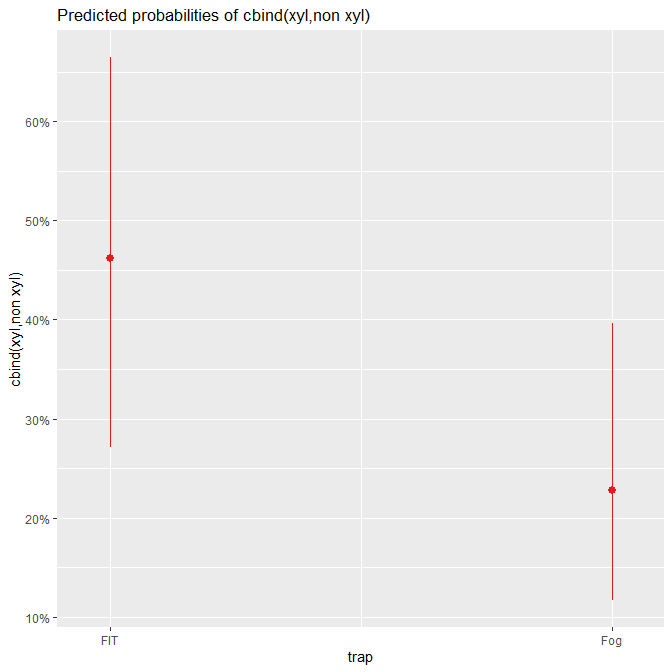


*Pinus individuals*


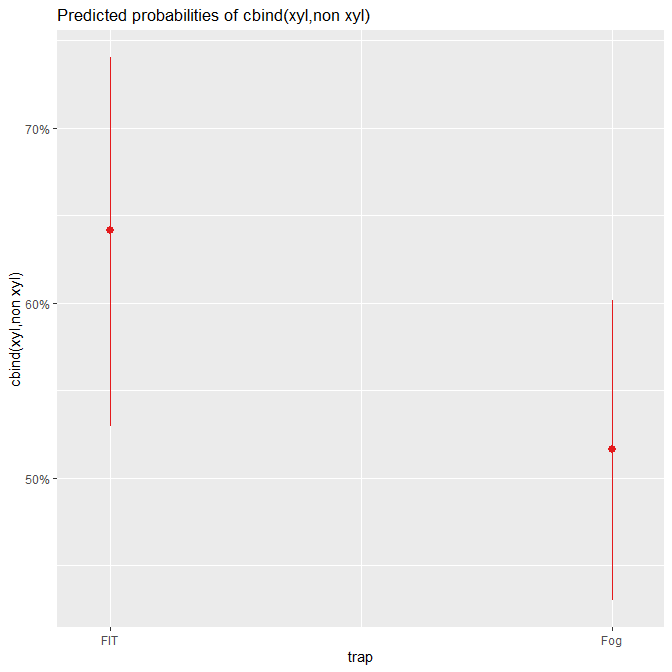

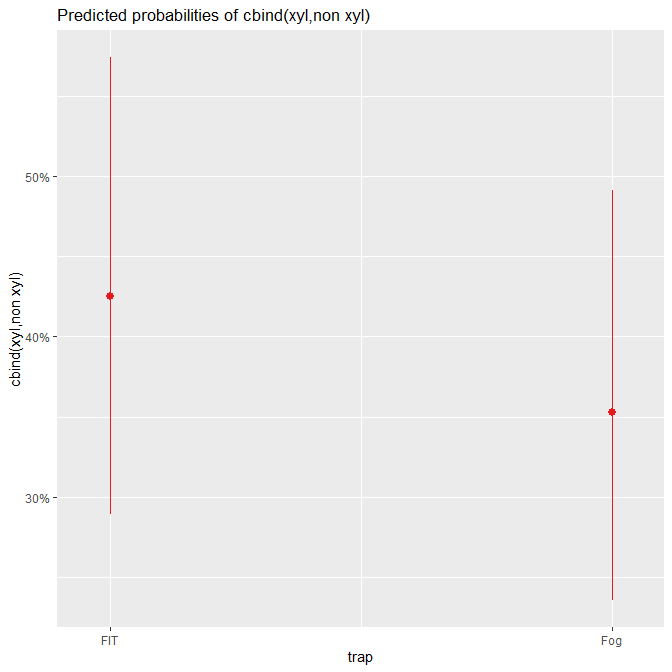


p = 0.001

*Fagus species*

*Pinus species*

p = 0.03

p = 0.02

ns

FIT

Fog

FIT

Fog

FIT

Fog

FIT

Fog

Xylobiont beetles (%)

**Table S1:** Species abundance distribution of the most common beetles in FITs and FOGGINGs on *Fagus sylvatica* (Fs) and *Pinus sylvestris* (Ps). Xylobiont species (Xylo) and feeding guild (Guild) assignments are also given. Scolytinae is a subfamily of Curculionidae; myc=mycetophages, xyl=xylophages, phy=phytophages, zoo=zoophages.


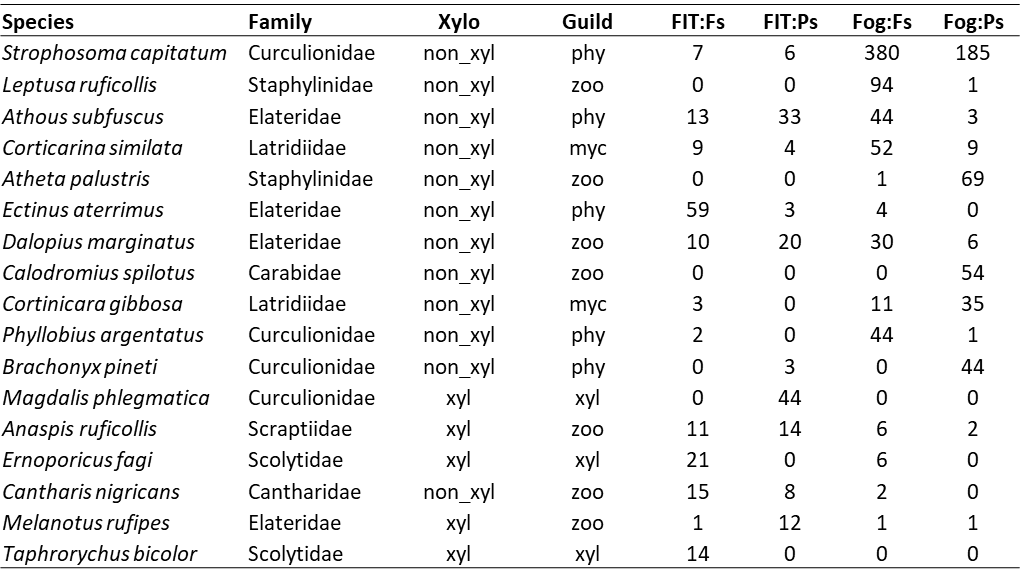


**Table S2:** A) Results of the adonis model show significances for the factors “Tree” (*F. sylvatica*, P. sylvestris), “Trap” (FIT, FOGGING), “SMI” (silvicultural management index) and their interaction term (trap * tree). The factor “Trap” explains most variability. The SMI is represented differently by FITs and FOGGINGs. B) Separate analyses for both tree species showed significant differences between sampling methods.


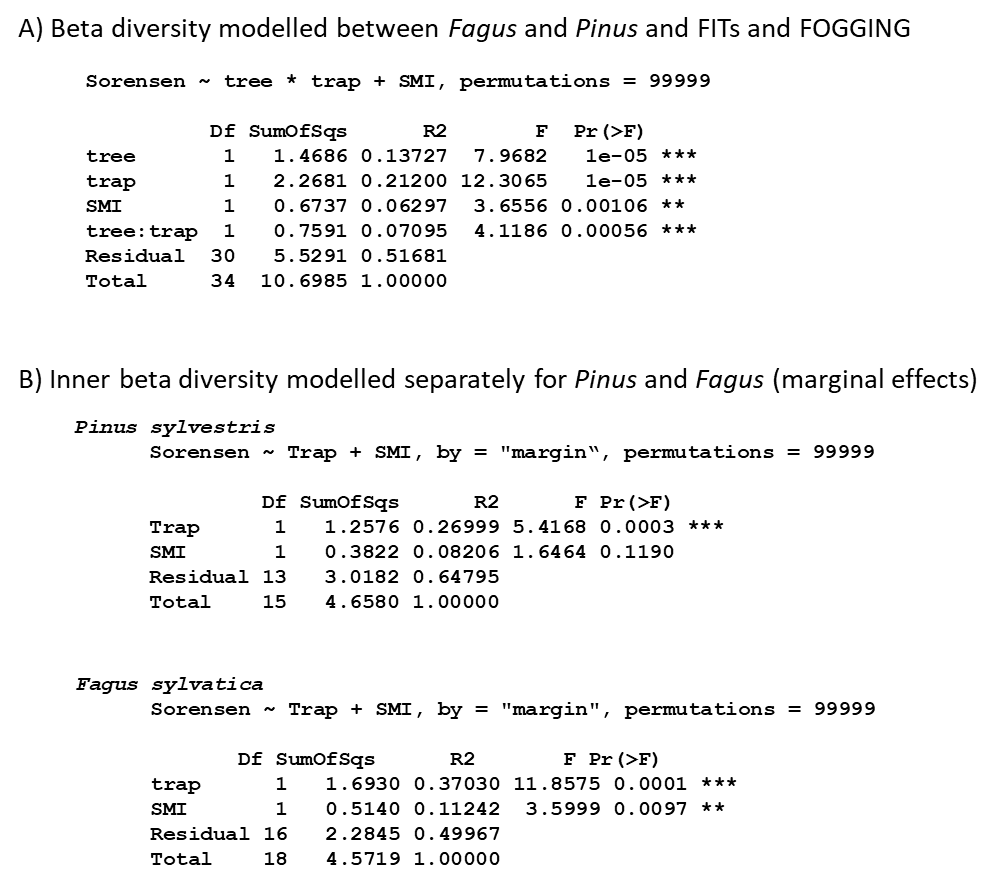


**Table S3:**  Result of quasibinomial mixed effect logistic regression: Singleton Proportion ~ Trap+ (1|plotID). The factor „Tree“ was not significant and therefore omitted from the model.


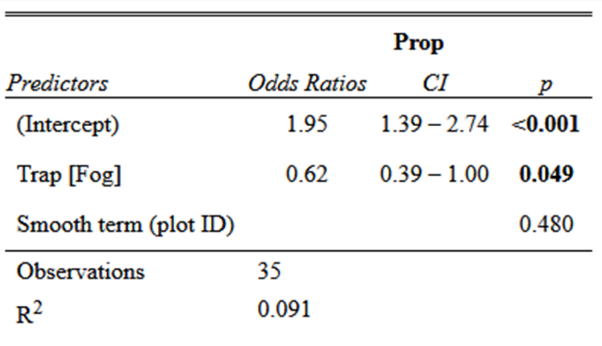


**Table S4:** Relative proportion and abundance of A) all Coleoptera and B) only xylobiont beetles collected by FITs and FOGGING from *Fagus sylvatica* (Fs) and *Pinus sylvestris* (Ps) in June. Shading according to relative proportions. Feeding guilds: myc=mycetophages, xyl=xylophages, zoo=zoophages, sap=saprophages, phy=phytophages.


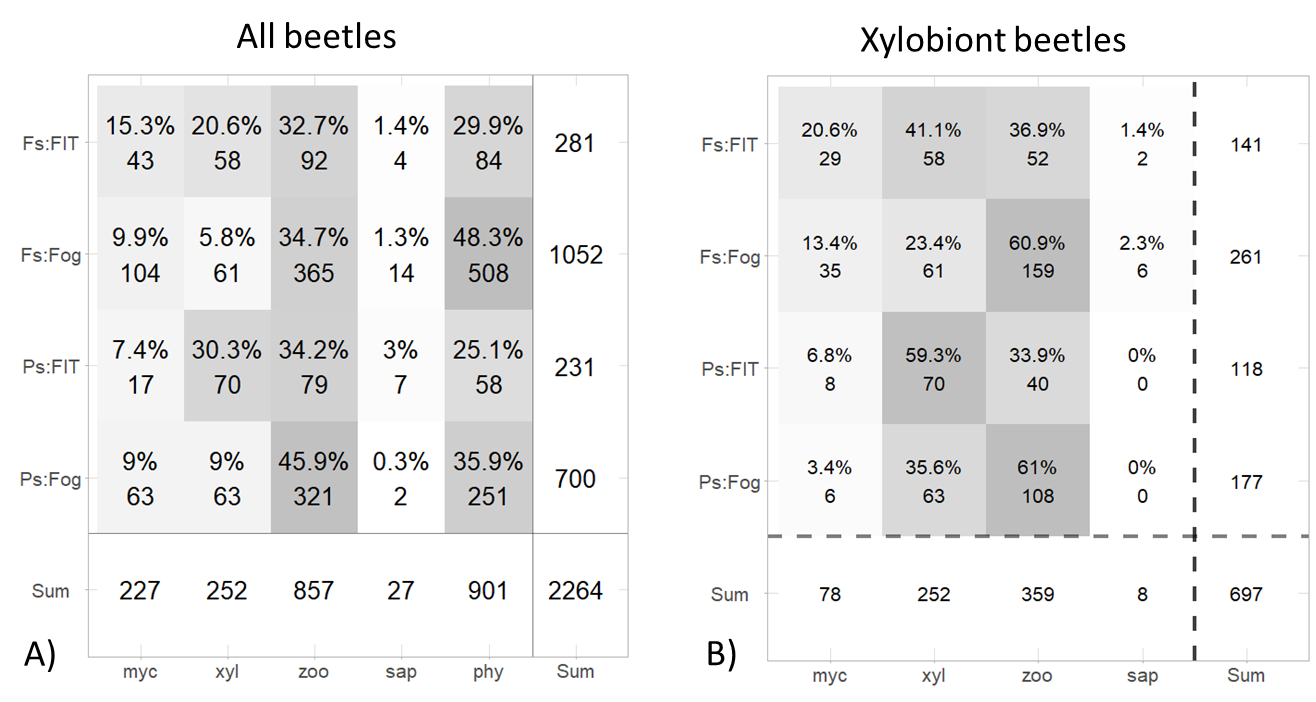


**Table S5:** Proportion of xylobiont beetles and species numbers in FITs and FOGGING collected from *Fagus sylvatica* and *Pinus sylvestris* in June.


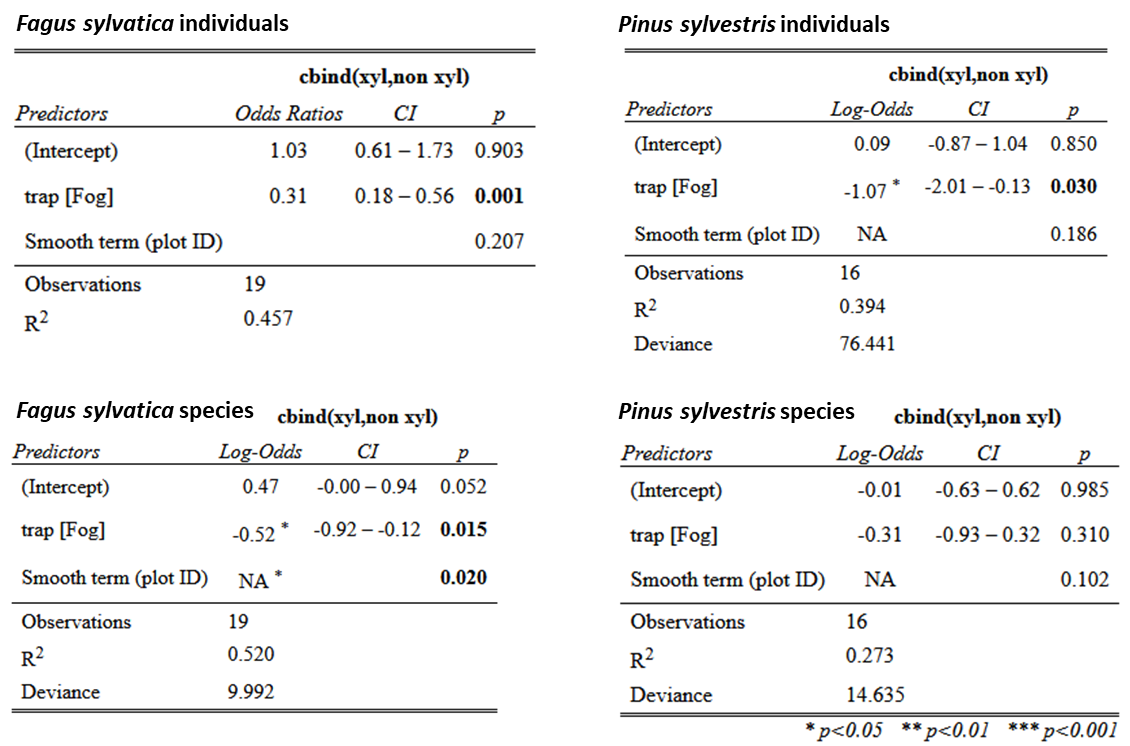

Supplement: Supplementary file 1 — Figure S1: Details on family, genus, species composition of beetle communities. Figure S2: UpSet plots for family, genus, species composition of beetle communities. Figure S3: Proportional on beetle distribution on the family level. Figure S4: Sample based rarefaction curves for beetles collected by FITs and FOGGING. Figure S5: Marginal effects of fitted mixed effect models for xylobiont beetles. Table S1: Species abundance distribution of the most common beetles in FITs and FOGGING. Table S2: Results of beta diversity modelling. Table S3: Result of modelling singleton proportions. Table S4: Guild composition of whole beetle communities and of xylobiont beetles alone. Table S5: Result of modelling xylobiont beetle proportions. Raw Data: All the required data are uploaded as the Supporting Information. [file ECE3-16-e73276-s001.docx]
